# Effect of anamorelin on body weight in patients with gastric cancer-associated cachexia: an observational study

**DOI:** 10.1007/s10120-025-01637-3

**Published:** 2025-07-08

**Authors:** Yoshitomo Yanagimoto, Kotaro Yamashita, Ryohei Kawabata, Takeshi Omori, Masaaki Motoori, Yujiro Nakahara, Yutaka Kimura, Haruna Furukawa, Takuro Saito, Kazuyoshi Yamamoto, Tsuyoshi Takahashi, Yukinori Kurokawa, Hidetoshi Eguchi, Yuichiro Doki

**Affiliations:** 1 Department of Gastroenterological Surgery, Toyonaka Municipal Hospital, Osaka, Japan; 2 Department of Gastroenterological Surgery, Osaka International Cancer Institute, Osaka, Japan; 3 Department of Gastroenterological Surgery, Osaka University Graduate School of Medicine, 2-2, Yamadaoka, Suita, Osaka 565-0871 Japan; 4 Department of Gastroenterological Surgery, Sakai City Medical Center, Osaka, Japan; 5 Department of Gastroenterological Surgery, Osaka International Medical and Science Center, Osaka Keisatsu Hospital, Osaka, Japan; 6 Department of Gastroenterological Surgery, Osaka General Medical Center, Osaka, Japan; 7 Department of Gastroenterological Surgery, Department of Surgery, Kindai Nara Hospital, Nara, Japan; 8 Department of Gastroenterological Surgery, Rinku General Medical Center, Osaka, Japan

**Keywords:** Anamorelin, Gastric cancer, Cancer cachexia, Body weight loss

## Abstract

**Background:**

In 2021, anamorelin, a ghrelin receptor agonist, was approved in Japan for cancer cachexia in select cancers, including gastric cancer. However, evidence regarding its efficacy and predictive factors in patients with gastric cancer remains lacking.

**Methods:**

This prospective observational study encompassed 229 patients with unresectable, advanced, or recurrent gastric cancer and cancer cachexia who received anamorelin from 2021 to 2023 at 25 institutions affiliated with Osaka University. Body weight change at 12 weeks was the primary endpoint. Appetite, food intake, treatment compliance, and adverse events comprised the secondary endpoints. Multivariable logistic regression analyses were employed for identifying weight gain predictors.

**Results:**

Of the 229 patients (median age, 73 years), 126 completed the 12-week follow-up. The median anamorelin administration duration was 62 days. The mean weight significantly increased from baseline to 4, 8, and 12 weeks (up to + 0.88 kg, p < 0.001). Moreover, appetite and food intake improved. Multivariable analysis identified baseline body mass index (BMI) < 20 kg/m^2^ and neutrophil-to-lymphocyte ratio (NLR) < 4.0 as independent predictors of significant weight gain at 12 weeks. Treatment was generally well tolerated, with a 41% completion rate; 59% of the participants discontinued mainly owing to disease progression.

**Conclusion:**

In patients with gastric cancer-related cachexia, anamorelin was associated with significant increases in body weight and improvements in appetite. Lower BMI and lower systemic inflammation (NLR < 4.0) were predictive of better response.

## Introduction

In 2021, anamorelin, a ghrelin receptor agonist, was approved in Japan for cancer cachexia treatment in patients with lung, gastric, colorectal, and pancreatic cancers [1, 2]. Its expected primary outcomes include the suppression of weight loss and skeletal muscle depletion, mainly through growth hormone secretion stimulation and appetite enhancement. Patients with gastric cancer are particularly predisposed to poor oral intake and are more susceptible to weight loss owing to complications and adverse events associated with therapeutic interventions, including chemotherapy and surgery [3]. Weight loss during gastric cancer treatment can cause a decline in activities of daily living and quality of life, potentially impacting treatment continuity [4, 5]. Therefore, in these patients, weight management represents a critical target for therapeutic interventions.

The clinical trial data used for the pharmaceutical approval of anamorelin primarily comprised cases of lung cancer, and data on gastrointestinal cancers, particularly gastric cancer, were lacking [1, 2]. However, postmarketing surveillance data have shown a 1.1-kg weight gain following a 12-week anamorelin treatment in patients with gastric cancer [6], suggesting its potential efficacy in cancer cachexia management in this population. Nevertheless, identifying appropriate candidates for intervention and predicting treatment efficacy before administration remain unclear, posing a significant clinical challenge.

This study aimed to evaluate the compliance, adverse events, appetite, and weight gain effects of anamorelin in patients with unresectable, advanced, or recurrent gastric cancer with cancer cachexia and analyze the treatment efficacy and its predictive factors.

## Materials and methods

### Study design and participants

This prospective observational study was conducted at 25 affiliated institutions of the Department of Gastroenterological Surgery, Osaka University, and enrolled cases from 2021 to 2023. Overall, this study included 229 patients diagnosed with unresectable or advanced/recurrent gastric cancer with cancer cachexia and treated with anamorelin. Cancer cachexia was defined based on the international consensus proposed by Fearon et al. [7]. The following were the inclusion criteria: (1) patients who had been pathologically diagnosed with gastric cancer; (2) those with cachexia who did not adequately respond to nutritional therapy or other treatments; (3) those who had experienced a weight loss of > 5% in the past 6 months; (4) those who met at least two of the following three conditions: fatigue or general malaise, generalized muscle weakness, and at least one of the following laboratory findings (C-reactive protein [CRP] ≥ 0.5 mg/dL, hemoglobin < 12 g/dL, or albumin < 3.2 g/dL); and (5) those who provided written informed consent for study participation. The following were the exclusion criteria: (1) patients with difficulty in oral food intake or impaired digestion and absorption; (2) those with a history of hypersensitivity to any component of the study drug; (3) those with congestive heart failure; (4) those with a history of myocardial infarction or angina pectoris; (5) those with severe conduction system disorders; (6) those receiving any of the following drugs: clarithromycin, indinavir, itraconazole, nelfinavir, saquinavir, telaprevir, voriconazole, ritonavir-containing formulations, or cobicistat-containing formulations); and (7) those with moderate or severe hepatic dysfunction (Child–Pugh classification B or C). Ethical approval for this study was obtained separately at each participating institution in accordance with institutional policies and the Declaration of Helsinki (IRB approval number: 20498–4).

### Outcomes

The primary endpoint was the change in body weight at 12 weeks after starting oral administration. Additionally, medication compliance, adverse events, and changes in appetite constituted the secondary endpoints. Body weight was measured using a standardized scale specified by each institution at baseline (before anamorelin administration) and at 4, 8, and 12 weeks following administration. Appetite was assessed using a patient self-reported questionnaire at the same time points: before anamorelin administration and at 4, 8, and 12 weeks thereafter. Appetite was assessed using a five-point scale: no appetite at all, slight appetite, moderate appetite, considerable appetite, and very good appetite. Food intake was evaluated using a four-point scale: uncertain, decreased food intake, no change in food intake, and increased food intake. Data on appetite and food intake were collected using a standardized checklist based on the FAACT, which was used uniformly across all participating institutions. The modified Glasgow Prognostic Score (mGPS) was categorized into three groups (0, 1, and 2) on the basis of previously reported criteria, using serum CRP levels (cutoff: 1.0 mg/dL) and serum albumin levels (cutoff: 3.5 g/dL) [8–10]. The neutrophil-to-lymphocyte ratio (NLR) was calculated by dividing the neutrophil count by the lymphocyte count. The lymphocyte-to-albumin ratio was calculated by dividing the lymphocyte count by the albumin level. The CRP-to-albumin ratio (CAR) was calculated by dividing the CRP level by the albumin level.

### Statistical analysis

Body weight and clinical data before and after anamorelin administration were compared. Cut-off values for continuous variables, such as BMI and NLR, were determined based on exploratory analysis of the current dataset. We assessed the distribution of each variable and its relationship with body weight change, and selected thresholds that most clearly distinguished the degree of weight loss. Although these cut-offs were not based on receiver operating characteristic (ROC) curve analysis or predefined criteria from previous literature, some of them, such as NLR < 4.0, are consistent with values reported in earlier studies [11]. This data-driven approach was intended to identify clinically meaningful trends for future validation. In this study, we analyzed data collected at 4, 8, and 12 weeks after the initiation of anamorelin, regardless of whether the medication was continued. To compare the proportions between the two groups, Pearson’s chi-square test was employed. To adjust for covariates, logistic regression analysis was performed. The t-test was used for comparing the mean values between the two groups. Analysis of covariance was conducted for covariate adjustment. Moreover, Cox proportional hazards modeling was employed to further evaluate the impact of the covariates. In the univariable and multivariable analyses, all factors presumed to be associated with weight change following anamorelin administration were included in the multivariable analysis, regardless of the results of the univariable analysis. Statistical analyses were performed using Statistical Package for the Social Sciences (version 29, IBM, Armonk, NY, USA). P < 0.05 was considered statistically significant.

### Role of the funding source

This study was conducted as a research project of the Japanese Gastric Cancer Association for the fiscal year 2023. The funder played no role in the study design, data collection, data analysis, data interpretation, or writing of the report. The corresponding author had full access to all the data in this study following study termination and had final responsibility for the decision to submit for publication.

## Results

### Patient characteristics

The baseline characteristics of the study population are summarized in Table 1. Overall, 229 patients with gastric cancer-associated cachexia were enrolled, with a median age of 73 (33–93) years. Among the participants, 156 (68%) and 73 (32%) were male and female, respectively. Most patients had advanced-stage gastric cancer, with 69 (30%) and 133 (58%) at stages III and IV, respectively, according to the Japanese Classification of Gastric Carcinoma 15th edition by the Japanese Gastric Cancer Association. At anamorelin administration initiation, 193 patients (84%) were receiving chemotherapy, whereas 36 patients (16%) were receiving the best supportive care. Regarding the number of chemotherapy lines, 120 (52%) and 109 (48%) patients were undergoing first-line and second-line or later treatments, respectively. A total of 124 patients (54%) had undergone gastrectomy before initiating anamorelin. The breakdown of surgical procedures was as follows: 53 patients underwent distal gastrectomy, 21 underwent proximal gastrectomy, 45 underwent total gastrectomy, and 5 underwent an unspecified type of gastrectomy. The median baseline body weight was 49.5 (29.0–76.0) kg, and the median body mass index (BMI) was 18.2 (12.4–28.4) kg/m^2^.

**Table 1:** Patient’s background

		N = 229
Age, years	Median (Range)	73 (33–93)
Sex	Male/Female	156/73
cStage	I/II/III/IV	9/18/69/133
Treatment at anamorelin administration initiation	Chemo/BSC	193/36
Number of chemotherapy lines	1st/2nd/3rd or later	120/35/38
Gastrectomy before anamorelin administration	performed/not performed	124/105
Gastrectomy type	DG/PG/TG/unknown	53/21/45/5
Weight, kg	Median (Range)	49.5 (29.0–76.0)
BMI, kg/m^2^	Median (Range)	18.24 (12.40–28.40)
Hemoglobin, g/dL	Median (Range)	10.6 (6.6–15.3)
Platelet count, × 10^3^ count/μL	Median (Range)	21.9 (7.5–57.2)
Serum albumin, g/dL	Median (Range)	3.3 (1.5–4.5)
Serum CRP, mg/dL	Median (Range)	0.61 (0.0–24.3)
WBC, count/μL	Median (Range)	5,240 (2,300–17,690)
Neutrophil count, count/μL	Median (Range)	3,305.5 (800–15,200)
Lymphocyte count, count/μL	Median (Range)	1,179 (11.5–4,100)

*BSC* best supportive care, *DG* distal gastrectomy, *PG* proximal gastrectomy, *TG* total gastrectomy, *BMI* body mass index, *CRP* C-reactive protein, *WBC* white blood cell

### Compliance and discontinuation of anamorelin administration

The compliance with anamorelin administration and the reasons for discontinuation are presented in Table 2. The overall compliance rate was 41%, with 94 patients completing the 12-week treatment course. However, 135 (59%) patients prematurely discontinued the treatment. The median duration of Anamorelin administration was 62 days. Disease progression (63 patients, 28%), adverse effects (15 patients, 7%), and patient refusal (12 patients, 5%) were the reasons for discontinuation. Adverse effects resulting in discontinuation included nausea (5 patients, 2%), hyperglycemia (3 patients, 1%), abdominal distension (3 patients, 1%), as well as fatigue, increased salivation, cholangitis, and decreased appetite, each occurring in one patient.

**Table 2:** Compliance with anamorelin administration and reasons for discontinuation

		N = 229
Anamorelin administration duration	Median (Range)	62 (1–629) days
Duration category	< 11 weeks/ ≥ 12 weeks	135 (59%)/94 (41%)
Reasons for discontinuation and median duration	Disease progression	63 cases, 27 (3–77) days
	Patient request	12 cases, 36 (1–70) days
	Nausea	5 cases, 5 (4–22) days
	Hyperglycemia	3 cases, 20 (3–76) days
	Abdominal distension	3 cases, 11 (6–79) days
	Fatigue	1 case, 50 days
	Increased salivation	1 case, 38 days
	Cholangitis	1 case, 17 days
	Decreased appetite	1 case, 1 day
	Conversion surgery	2 cases, 35 (14–42) days
	Unknown	43 cases, 41 (2–79) days

### Changes in body weight following anamorelin administration

**Fig. 1:**
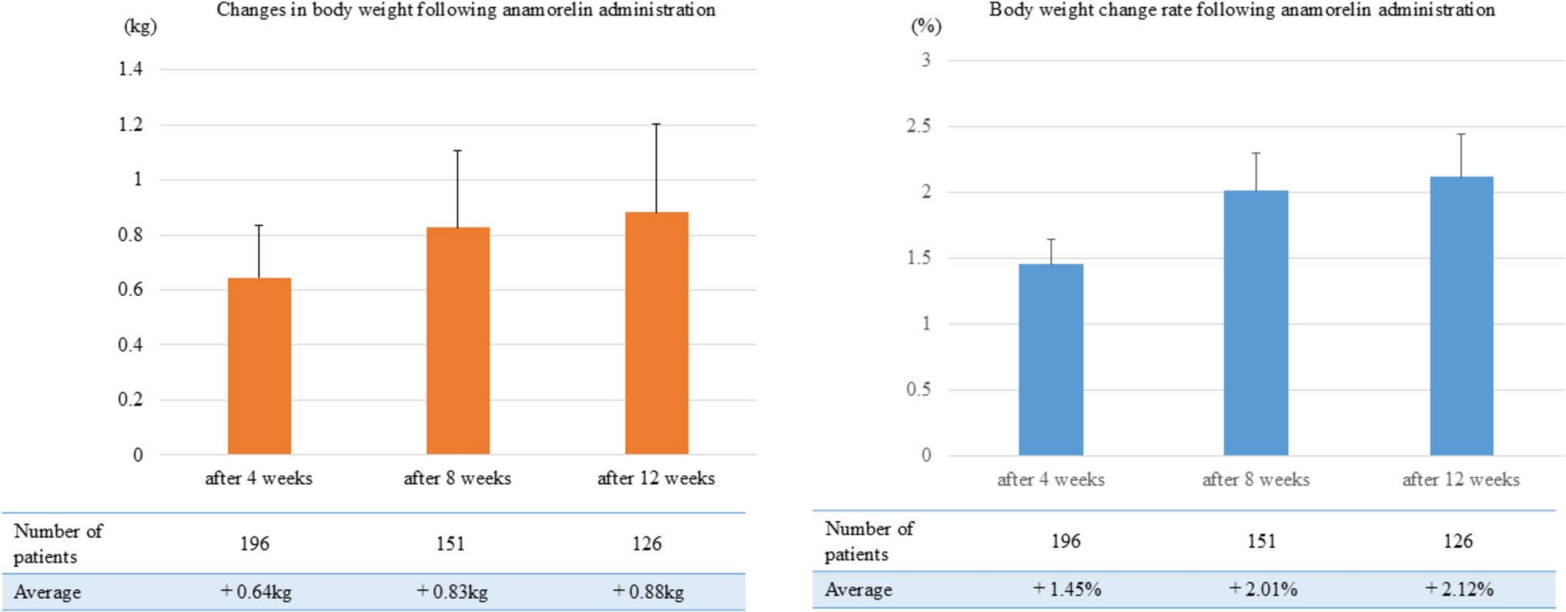
Changes in body weight following anamorelin administration. This figure illustrates the changes in body weight and the body weight change rate over time following anamorelin administration. Body weight is significantly increased after 4, 8, and 12 weeks of treatment. Furthermore, the body weight change rate exhibits a positive trend, with most patients experiencing progressive weight gain over time

The changes in body weight following anamorelin administration are illustrated in Fig. 1. After 4 weeks of treatment, body weight significantly increased by an average of 0.64 kg (1.45%) (p < 0.001). At 8 weeks, the mean body weight increased by 0.83 kg (2.01%) (p < 0.001); at 12 weeks, it further increased by 0.88 kg (2.12%) (p < 0.001). Of the 126 patients whose body weight data were collected at 12 weeks following treatment initiation, 39 (31%) demonstrated a ≥ 5% weight gain, whereas 44 (35%) exhibited a 0%–5% weight gain. In contrast, 43 patients (33%) experienced weight loss.

### Changes in appetite and food intake

**Fig. 2:**
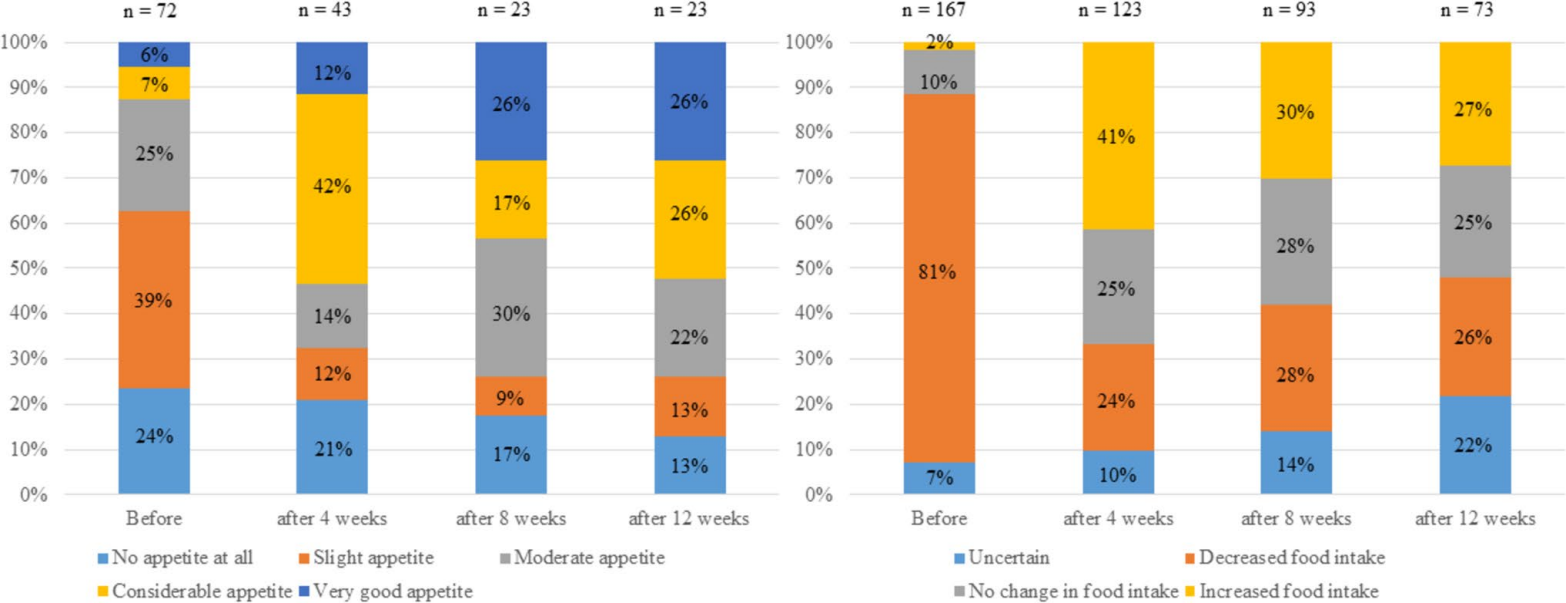
Changes in appetite and food intake following anamorelin administration**.** This figure presents the changes in appetite and food intake at baseline and after 4, 8, and 12 weeks of anamorelin administration. The proportion of patients with enhanced appetite has increased over time, whereas the percentage of those with no appetite has decreased. Similarly, food intake demonstrates a positive trend, with a higher proportion of patients reporting increased intake over time

The changes in appetite and food intake following anamorelin administration are depicted in Fig. 2. Regarding appetite, at baseline, 24% of the patients reported having no appetite at all, and 39% had only slight appetite. After 4 weeks of treatment, appetite improvement was observed, with increased proportion of patients reporting considerable (42%) or very good appetite (12%). This trend continued over time, with 26% of the patients reporting very good appetite at both 8 and 12 weeks. Additionally, the proportion of patients with no appetite at all decreased from 24% at baseline to 13% after 12 weeks. Furthermore, food intake exhibited a positive trend over time. At baseline, 81% of the patients reported decreased food intake, with only 10% showing no change and 2% reporting increased intake. After 4 weeks of treatment, the proportion of patients with decreased food intake significantly decreased to 24%, whereas increased food intake was reported in 41%. At 12 weeks, 27% of the patients demonstrated increased food intake, whereas only 26% continued to report decreased food intake.

### Weight gain-associated factors

The results of the univariable and multivariable analyses of weight gain-related factors at 4, 8, and 12 weeks following anamorelin administration are presented in Table 3. In the analysis at 4 weeks following administration, neither univariable nor multivariable analysis identified any significant factors associated with weight gain. Regarding weight gain at 8 weeks, univariable analysis did not identify any significant factors. However, in the multivariable analysis, including all potential factors, a baseline BMI of < 20 kg/m^2^ (adjusted odds ratio [OR], 3.14; 95% confidence interval [CI], 1.27–7.79; p = 0.013) and serum hemoglobin levels of ≥ 10 g/dL (adjusted OR, 2.62; 95% CI, 1.16–5.94; p = 0.021) were identified as significant factors. Regarding weight gain at 12 weeks, univariable analysis revealed a baseline BMI of < 20 kg/m^2^ (adjusted OR, 2.44; 95% CI, 1.09–5.44; p = 0.029) and an NLR of < 4.0 (adjusted OR, 3.00; 95% CI, 1.27–7.07; p = 0.012) as significant factors. These factors remained significant in the multivariable analysis, with a baseline BMI of < 20 kg/m^2^ (adjusted OR, 3.89; 95% CI, 1.33–11.35; p = 0.013) and an NLR of < 4.0 (adjusted OR, 3.51; 95% CI, 1.12–11.02; p = 0.031) being identified as independent predictors of weight gain.

**Table 3:** Univariable and multivariable analyses of weight gain following anamorelin administration

		OR	95% CI	P-value	OR	95% CI	P-value
*Analysis of weight gain after 4 weeks of administration*		*Univariable analysis*			*Multivariable analysis*		
Age	≥ 76 years	1.00	0.55–1.81	0.99	1.03	0.53–1.98	0.94
Sex	Male	1.51	0.79–2.89	0.21	1.69	0.82–3.49	0.15
Number of chemotherapy lines	1st line	1.55	0.85–2.82	0.15	1.64	0.86–3.12	0.13
Gastrectomy before anamorelin administration	Performed	1.25	0.69–2.26	0.47	1.05	0.53–2.10	0.88
BMI	≤ 20 kg/m^2^	1.36	0.71–2.58	0.35	1.07	0.52–2.22	0.86
Hb	≥ 10 g/dL	0.71	0.37–1.34	0.29	0.88	0.44–1.76	0.72
mGPS	0	1.06	0.57–2.00	0.85	1.00	0.46–2.15	0.99
NLR	≤ 4.0	1.06	0.56–2.02	0.85	1.42	0.64–3.16	0.38
LAR	≤ 300	1.61	0.84–3.08	0.15	1.83	0.83–4.02	0.14
CAR	≤ 0.3	1.02	0.52–1.98	0.96	0.77	0.35–1.67	0.50
*Analysis of weight gain after 8 weeks of administration*		*Univariable analysis*			*Multivariable analysis*		
Age	≥ 76 years	1.10	0.56–2.17	0.78	1.72	0.76–3.86	0.19
Sex	Male	1.35	0.65–2.79	0.42	1.18	0.49–2.83	0.71
Number of chemotherapy lines	1st line	1.17	0.59–2.31	0.65	1.69	0.77–3.68	0.19
Gastrectomy before anamorelin administration	Performed	0.86	0.44–1.69	0.66	0.54	0.23–1.26	0.15
BMI	≤ 20 kg/m^2^	1.97	0.97–3.99	0.061	3.14	1.27–7.79	0.013
Hb	≥ 10 g/d	1.66	0.83–3.35	0.15	2.62	1.16–5.94	0.021
mGPS	0	1.31	0.64–2.70	0.46	1.07	0.44–2.64	0.88
NLR	≤ 4.0	1.43	0.67–3.05	0.35	1.08	0.40–2.93	0.88
LAR	≤ 300	0.77	0.39–1.55	0.47	0.89	0.36–2.22	0.80
CAR	≤ 0.3	1.69	0.78–3.67	0.18	1.41	0.56–3.52	0.46
*Analysis of weight gain after 12 weeks of administration*		*Univariable analysis*			*Multivariable analysis*		
Age	≥ 76 years	1.32	0.61–2.85	0.48	1.76	0.70–4.47	0.23
Sex	Male	1.11	0.50–2.47	0.79	1.16	0.42–3.22	0.77
Number of chemotherapy lines	1st line	1.38	0.64–3.00	0.42	2.13	0.84–5.38	0.11
Gastrectomy before anamorelin administration	Performed	1.05	0.50–2.22	0.90	0.46	0.17–1.25	0.13
BMI	≤ 20 kg/m^2^	2.44	1.09–5.44	0.029	3.89	1.33–11.35	0.013
Hb	≥ 10 g/dL	1.46	0.66–3.23	0.35	1.88	0.73–4.86	0.19
mGPS	0	1.57	0.70–3.52	0.27	1.83	0.66–5.08	0.25
NLR	≤ 4.0	3.00	1.27–7.07	0.012	3.51	1.12–11.02	0.031
LAR	≤ 300	0.55	0.25–1.21	0.14	1.10	0.38–3.22	0.86
CAR	≤ 0.3	1.08	0.48–2.46	0.85	0.75	0.26–2.14	0.59

*BMI* body mass index, *Hb* hemoglobin, *mGPS* modified Glasgow Prognostic Score, *NLR* neutrophil-to-lymphocyte ratio, *LAR* lymphocyte-to-albumin ratio, *CAR* C-reactive protein-to-albumin ratio

### Stratified analysis of the weight gain

**Fig. 3:**
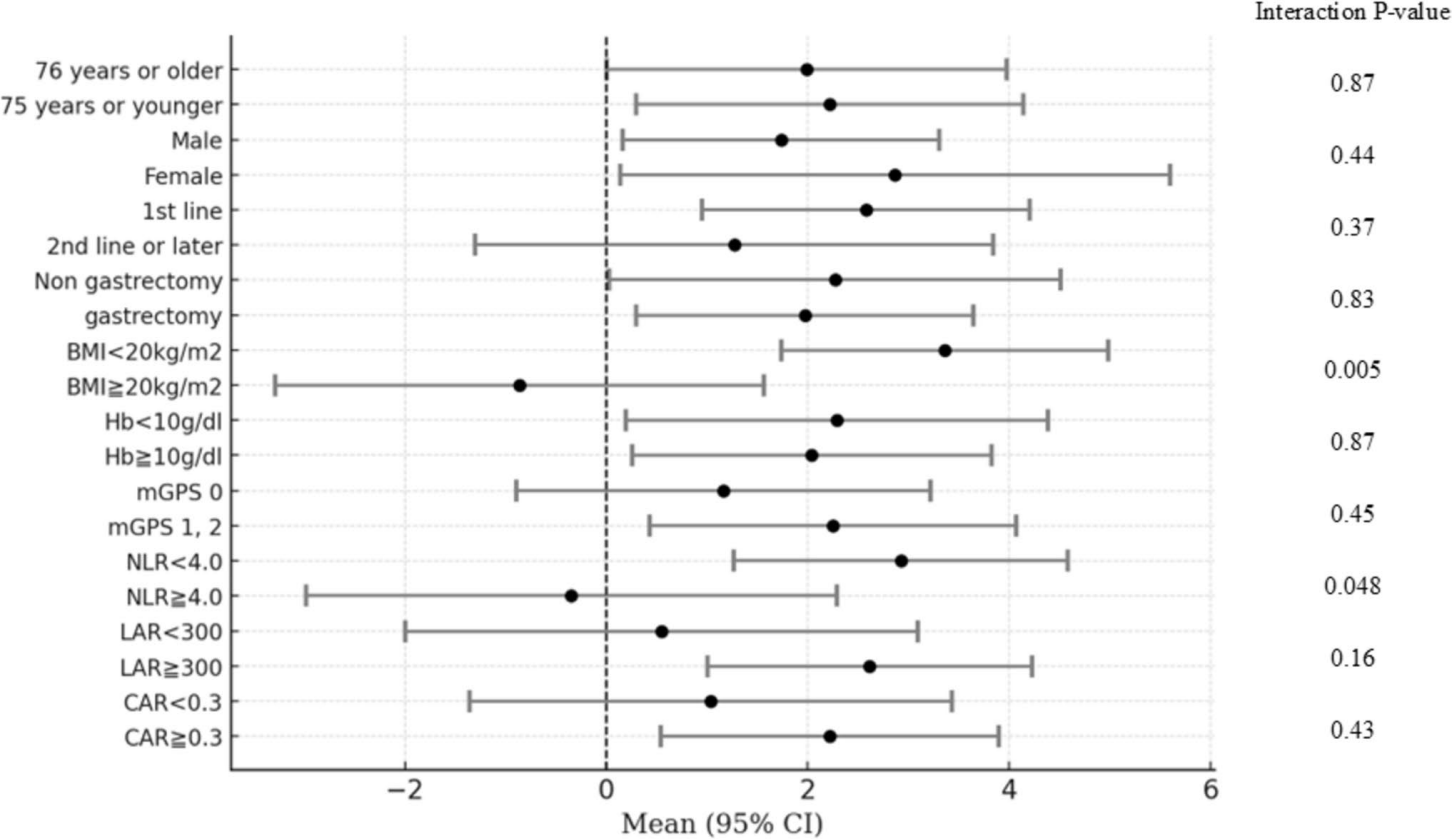
Stratified analysis of the weight gain rate after 12 weeks of anamorelin administration. This figure illustrates the stratified analysis of weight gain rates after 12 weeks of anamorelin administration. To identify potential predictive factors for treatment efficacy, subgroup analyses are performed on the basis of baseline characteristics. (*BMI* body mass index; *Hb* hemoglobin; *mGPS* modified Glasgow Prognostic Score; *NLR* neutrophil-to-lymphocyte ratio; *LAR* lymphocyte-to-albumin ratio; *CAR* C-reactive protein-to-albumin ratio; *CI* confidence interval)

A stratified analysis of the weight gain rates following 12 weeks of anamorelin administration is shown in Fig. 3. Among patients with a baseline BMI of < 20.0 kg/m^2^, the mean weight change rate (95% CI) was 3.36% (1.74–4.98), whereas among those with a baseline BMI of ≥ 20.0 kg/m^2^, it was − 0.86% (− 3.29 to 1.57). A significant interaction was observed (P = 0.005). Furthermore, the mean weight change rate (95% CI) was 2.92% (1.27–4.58) among patients with an NLR of < 4.0, whereas it was − 0.35% (− 2.98 to 2.29) among those with an NLR of ≥ 4.0. A statistically significant interaction was observed (P = 0.048).

## Discussion

In this observational study conducted at the Department of Gastroenterological Surgery, Osaka University, we evaluated the clinical efficacy of anamorelin in patients with gastric cancer-associated cachexia, successfully collecting data from 229 cases. Since the approval of anamorelin for the management of cancer cachexia, several clinical studies have investigated its efficacy. However, studies specifically focusing on patients with gastric cancer have been limited to post-marketing surveillance data provided by pharmaceutical companies, and to date, no detailed analyses of predictive factors influencing its efficacy have been reported [12–16]. In the present study, we demonstrated that oral administration of anamorelin in patients with gastric cancer-associated cachexia can be performed with relative safety. Moreover, a 12-week treatment period resulted in a significant and measurable increase in body weight. Notably, patients with lower baseline body mass index (BMI) exhibited a greater likelihood of achieving weight gain following treatment.

A previous postmarketing surveillance study reported a mean body weight gain of 1.1 kg following a 12-week course of anamorelin treatment [6]. Although that study included follow-up data for 1,076 patients with gastric cancer, only 294 patients had evaluable data at the 12-week time point, and the median duration of anamorelin administration was limited to 29 days. In contrast, in the present study, 126 of the 229 enrolled patients were successfully followed for 12 weeks, with a median treatment duration of 62 days and a mean body weight gain of 0.88 kg. Although direct comparisons between the two studies are inherently challenging due to differences in disease stage and treatment regimens, our findings demonstrated no clear association between improved treatment adherence and enhanced therapeutic benefits of anamorelin. These observations suggest that, in patients with gastric cancer-related cachexia, identifying predictive factors for anamorelin efficacy prior to treatment initiation may be of critical importance.

Although studies solely focusing on gastric cancer-related cachexia have not been conducted, several studies have investigated the efficacy of anamorelin in other malignancies, including pancreatic, colorectal, and non-small-cell lung cancers. Tsunematsu et al., in a study of 31 patients with pancreatic cancer, reported that a low CAR was a favorable predictive factor for the efficacy of anamorelin after 12 weeks [12]. Similarly, other studies involving patients with gastric, colorectal, pancreatic, and lung cancers have indicated that a lower mGPS, also based on serum CRP and albumin levels, was associated with a better treatment response [13, 14]. Moreover, Fujii et al. noted that patients with relatively preserved nutritional status, as indicated by a low Controlling Nutritional Status score calculated from serum albumin, total lymphocyte count, and total cholesterol levels, demonstrated significantly greater benefits from anamorelin across four cancer types for which anamorelin is approved for insurance coverage [15]. Although these studies included small sample sizes and diverse populations of patients with cancer, they collectively suggest that anamorelin can be more effective in patients who have not yet developed marked systemic inflammation or severe malnutrition. Consistent with these findings, our analysis revealed that patients with a high NLR, a systemic inflammation marker, were less likely to benefit from anamorelin, supporting the hypothesis that the systemic inflammatory burden may negatively affect the drug’s efficacy. In this study, mGPS and CAR—both calculated from serum albumin and CRP—were not identified as clear predictive markers of the efficacy of anamorelin. One possible explanation is that the eligibility criteria for anamorelin include albumin and CRP levels, and many patients in this cohort had abnormal values in these parameters at baseline, potentially attenuating the predictive utility of mGPS and CAR. Furthermore, hemoglobin levels ≥ 10 g/dL were identified as a significant factor associated with weight gain at 8 weeks after initiating anamorelin. Although no prior studies have reported a direct association between hemoglobin levels and the weight-increasing effect of anamorelin, previous literature has suggested that hemoglobin may be related to the progression and prognosis of cancer cachexia [7]. Furthermore, since hemoglobin reflects systemic status including nutritional condition, it is plausible that patients with hemoglobin ≥ 10 g/dL, indicating relatively preserved systemic condition, experienced greater weight gain. In patients with elevated systemic inflammation and malnutrition, the attenuated efficacy of anamorelin may be attributed, in part, to the underlying pathophysiological mechanisms. Cancer-related systemic inflammation activates pro-inflammatory cytokines, including interleukin (IL)-1, IL-6, and tumor necrosis factor alpha, which suppress appetite by acting on the hypothalamus and contribute to catabolic processes, including muscle degradation and lipolysis [17, 18]. These cytokines also disrupt ghrelin signaling, potentially weakening the appetite-stimulating effects of anamorelin [19]. Furthermore, the anabolic response to growth hormone and IGF-1 may be impaired by chronic inflammation, further limiting the efficacy of anamorelin in promoting weight or muscle gain [19]. Moreover, malnutrition and poor protein reserves may reduce the body’s capacity to respond to anabolic stimuli, further reducing the therapeutic potential of anamorelin.

Interestingly, the present study suggests that patients with a BMI of ≥ 20 kg/m^2^ were less likely to benefit from anamorelin. Although the median BMI in our cohort was 18.24 kg/m^2^, a subset of patients exhibited relatively higher BMI values. In these patients, baseline oral intake might have been sufficiently maintained, thereby limiting the appetite-stimulating effects of anamorelin. Notably, additional analyses of corporate clinical trial data related to the development of anamorelin have also reported greater efficacy in patients with BMI values below 20 kg/m^2^, which is consistent with the findings of our study [20]. To date, no studies have specifically identified an optimal BMI cutoff value for predicting the efficacy of anamorelin, and no established consensus currently exists. Nevertheless, early intervention during the pre-cachexia phase, prior to significant weight loss and malnutrition, is generally considered advantageous [7]. Based on the data from our study, a BMI of 20 kg/m^2^ may represent a potential threshold for selecting gastric cancer patients who are more likely to respond favorably to anamorelin therapy.

At the outset of this study, we hypothesized that patients who had undergone gastrectomy may derive greater benefits from anamorelin treatment than those with an intact stomach. This assumption was based on anamorelin’s mechanism of action as a ghrelin receptor agonist and the fact that circulating ghrelin levels are significantly decreased following gastrectomy [21]. Consequently, we expected that patients without a history of gastrectomy, who may demonstrate chronically elevated ghrelin levels due to cancer cachexia, would be less responsive to additional ghrelin receptor stimulation [22]. However, our findings showed that prior gastrectomy and body weight gain from anamorelin administration were not significant associated. Although this study did not measure serum ghrelin levels, previous studies have suggested that ghrelin levels can gradually recover over time in patients who had distal gastrectomy but not in those who underwent total gastrectomy [21]. This finding may partly explain the discrepancy between our hypothesis and the observed results. To clarify this issue, further prospective studies integrating serum ghrelin measurements are warranted.

This study had several limitations. First, we could not accurately evaluate skeletal muscle mass and muscle strength. Although the study protocol initially included plans to collect these data, performing it in the real-world clinical setting of an observational study involving patients with gastric cancer-related cachexia was challenging. Although previous clinical trials have reported that anamorelin increases skeletal muscle mass, no significant improvement in muscle strength was observed [2]. Therefore, future research focusing on the effects of anamorelin on muscle strength in patients with gastrointestinal cancer is needed. Furthermore, we attempted to collect data on nutritional markers such as rapid turnover proteins as surrogate indicators of skeletal muscle mass; however, the number of cases in which such data were available was too small to perform a reliable analysis. Second, as this study is an observational investigation involving gastric cancer patients treated with anamorelin, we were not able to perform a comparative analysis with a control group. To account for potential confounding factors inherent to this study design, we conducted multivariable analyses as a means of adjustment; however, we acknowledge that residual confounding may still exist. Furthermore, it is important to interpret the findings of this study in the context that anamorelin treatment was initiated based on the discretion of individual clinicians, and that a considerable proportion of patients were unable to complete 12 weeks of treatment, mainly due to disease progression. To address this, we are currently conducting a randomized controlled trial within our group to assess the efficacy of anamorelin in gastric cancer-related cachexia, and the results of that study are eagerly awaited. The last limitation of this study is that the analysis of treatment efficacy at 12 weeks after initiating anamorelin was restricted to patients who completed the 12-week follow-up. This factor should be taken into consideration when interpreting the study findings.

In conclusion, we analyzed 229 patients with gastric cancer-related cachexia who received anamorelin and confirmed that the treatment led to body weight gain. The NLR and BMI were identified as potential predictive factors for treatment efficacy. Active consideration of anamorelin therapy is warranted for patients likely to benefit from treatment, and clinical benefits beyond weight gain and skeletal muscle increase should be explored in future research.
